# Genetic diversity of *Enterocytozoon bieneusi* in 1099 wild animals and 273 imported pastured donkeys in northern China

**DOI:** 10.1186/s13071-025-06739-6

**Published:** 2025-03-13

**Authors:** Ziqi Wang, Nannan Cui, Jia Zhang, Zhixian Jiang, Ruiqi Song, Wenbo Tan, Meihua Yang, Sándor Hornok, Yuanzhi Wang

**Affiliations:** 1 Key Laboratory for Prevention and Control of Emerging Infectious Diseases and Public Health Security of the XPCC, School of Medicine, Shihezi University, Shihezi, 832002 Xinjiang Uygur Autonomous Region People’s Republic of China; 2 NHC Key Laboratory of Prevention and Treatment of Central Asia High Incidence Diseases, Shihezi University, Shihezi, 832003 Xinjiang Uygur Autonomous Region People’s Republic of China; 3 Department of Forest, College of Agriculture, Shihezi University, Shihezi, 832002 Xinjiang Uygur Autonomous Region People’s Republic of China; 4 Department of Parasitology and Zoology, University of Veterinary Medicine, Budapest, Hungary

**Keywords:** *Enterocytozoon bieneusi*, Wildlife, Genetic diversity, *ITS*

## Abstract

**Background:**

*Enterocytozoon bieneusi* is the most frequently detected microsporidian species in humans, wildlife and domestic animals. In northern China, to the best of our knowledge, no information on *E. bieneusi* infection has been reported in wild animals. The aim of the present study was to survey the occurrence of and genetically characterize *E. bieneusi* from a broad spectrum of vertebrate species in this region.

**Methods:**

A total of 1372 small intestine or fecal specimens were collected from 1019 mammals, 121 reptiles and 232 birds in Xinjiang Uygur Autonomous Region (XUAR) and Inner Mongolia Autonomous Region (IMAR), northern China. Each animal species was identified according to morphological characteristics and amplification of mitochondrial genes. Genotype analysis of *E. bieneusi* was performed by amplifying the internal transcribed spacer (*ITS*) region.

**Results:**

A total of 68 wild animal species were identified, including 34 mammal species, six reptile species and 28 bird species. The average rate of infection with *E. bieneusi* was 9.7% (133/1372 specimens). Twelve genotypes of *E. bieneusi*, including BEB6, CHG7, D, E, EbpD, horse1, MWC_d1, NCF2, NCF6, PL14, SN45 and XJHT4, were identified in specimens from XUAR, IMAR and Kyrgyzstan. Phylogenetically, these genotypes belonged to four groups, namely Group 1, Group 2, Group 12 and Group 14.

**Conclusions:**

To our knowledge, this study reports for the first time *E. bieneusi* genotype NCF2 in marbled polecats (*Vormela peregusna*), genotype NCF6 in red foxes (*Vulpes vulpes*), genotype D in grey wolf (*Canis lupus*), genotypes CHG7, horse1 and PL14 in rodents and genotypes MWC_d1, PL14 and SN45 in wild birds*.* The results also indicate that genotypes horse1, NCF2 and NCF6 were acquired either by the fecal–oral transmission route or during predator–prey interaction.

**Graphical Abstract:**

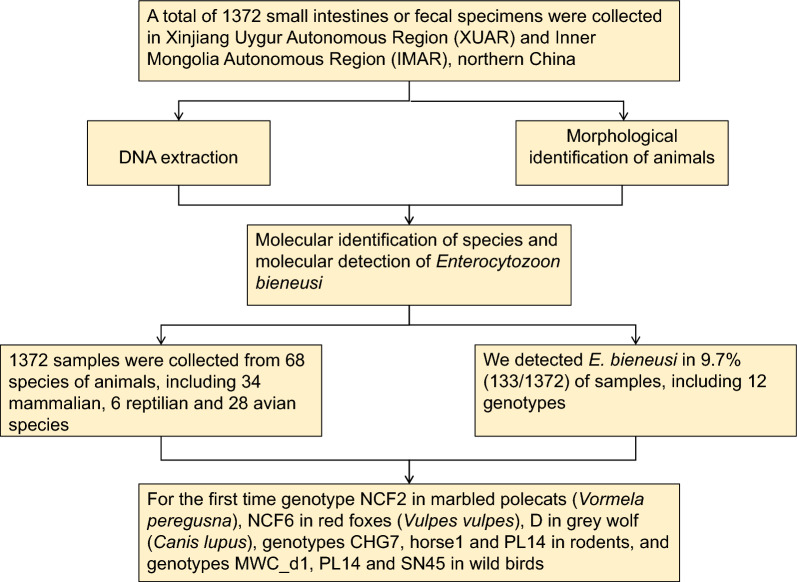

**Supplementary Information:**

The online version contains supplementary material available at 10.1186/s13071-025-06739-6.

## Background

*Enterocytozoon bieneusi*, a fungus-like protist parasite, is the most frequently detected microsporidian species causing microsporidiosis, accounting for > 90.0% of human microsporidiosis cases. The main clinical manifestation of *E. bieneusi* infection is diarrhea [1]. The life-cycle stages of *E. bieneusi* include schizonts, sporonts and spores. Infectious spores can usually be acquired from the environment by multiple host species, but some genotypes of *E. bieneusi* are host specific [2]. It has been isolated from a variety of host taxa, including humans, companion animals, livestock and wildlife. Since mature spores are shed in the host’s feces, the transmission routes of this pathogen may involve person-to-person contact as well as water-borne or food-borne infection, especially in developing countries [3]. Due to the variety of transmission routes, the sylvatic epidemiological cycle, which involves wild animal hosts such as carnivores and rodents, can pose a significant risk factor to the health of humans and domestic animals living nearby.

*Enterocytozoon bieneusi* genotypes are usually identified and classified by PCR and sequence analysis of the internal transcribed spacer region (*ITS*) of nuclear ribosomal DNA (rRNA) [4]. To date, approximately 600 distinct genotypes of *E. bieneusi* have been recorded in humans and approximately 170 distinct genotypes animal species. Among these, 49 genotypes have been found both in humans and animals [5].

In the southwestern and central regions of China, 361 *E. bieneusi* genotypes have been confirmed [6]. These genotypes were assigned to 14 major genetic groups, denoted as Group 1 to 14. Interestingly, genotypes in Groups 1 and 2 were found to be zoonotic, while genotypes in Groups 3 to 14 were restricted to specific hosts, as exemplified by genotype CD5 that is only detected in dogs [7, 8].

Xinjiang Uygur Autonomous Region (XUAR) and Inner Mongolia Autonomous Region (IMAR), located in northern China, cover 1,6749 and 1,1830 million km^2^, respectively [9]. The multiple natural landscapes (e.g. deserts, alpine meadows, forests and wetlands) and vast area of these regions provide suitable habitats for an abundance of terrestrial wildlife, including mammals and birds. These regions also have several endemic species, such as the Yarkand hare (*Lepus yarkandensis*)*.* To evaluate the zoonotic potential of the isolates at the genotype level in XUAR and IMAR, the aim of this study was to explore the genotypes and phylogenetic groups of *E. bieneusi* detected in samples from 1084 wild animals, 15 zoo inhabitants and 273 pastured donkeys (*Equus asinus*) imported from Kyrgyzstan.

## Methods

### Animal fecal sample collection

**Fig. 1:**
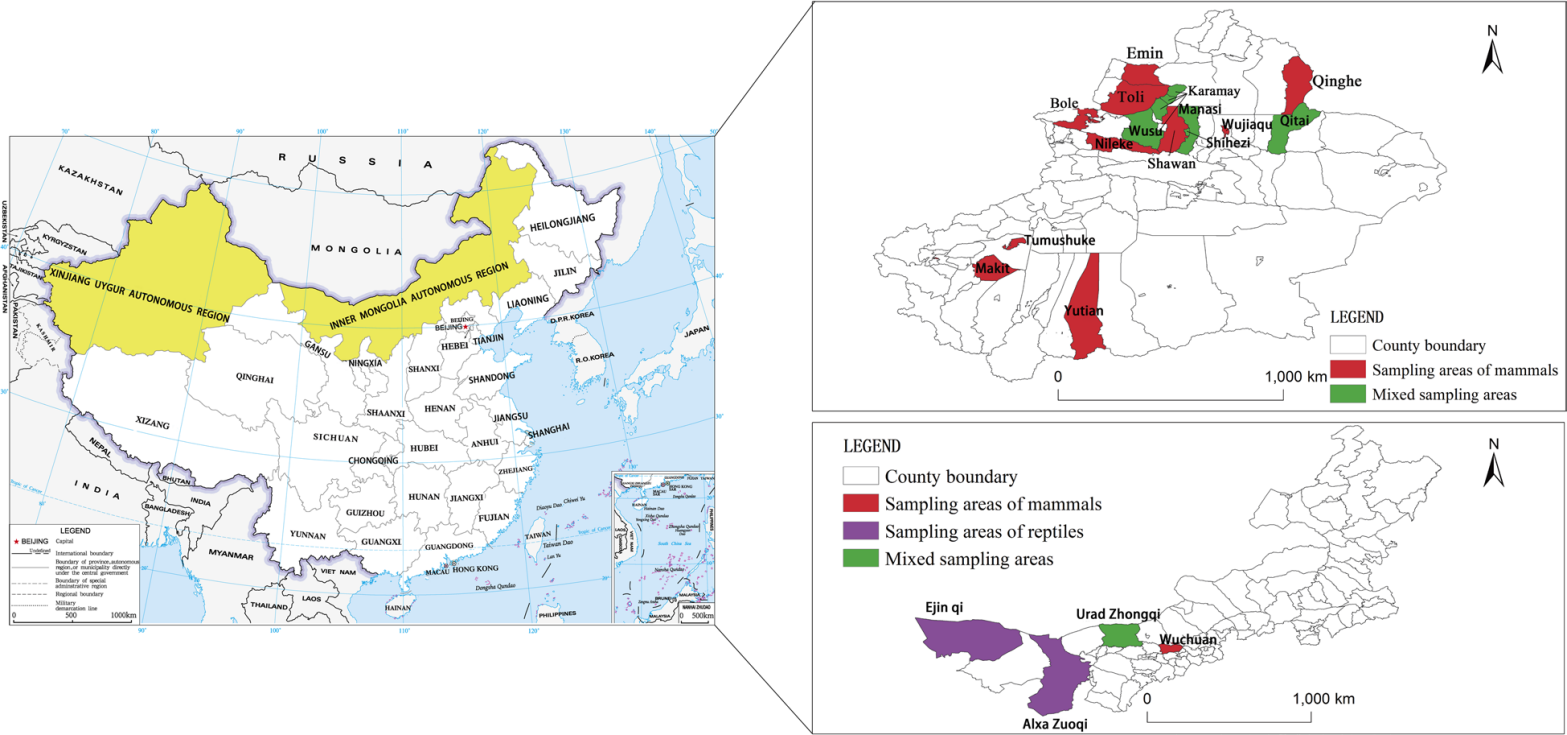
Locations where samples were collected for this study

During 2015–2024, intestinal samples from 273 pastured donkeys imported from Kyrgyzstan and part of the intestine or fecal samples from 1099 wild animals were collected in XUAR and IMAR, including 610 wild rodents, 81 Mongolian pikas (*Ochotona pallasi*), one Yarkand hare (*Lepus yarkandensis*), two common shrews (*Sorex araneus*), three Przewalski’s gazelles (*Procapra przewalskii*), 16 red foxes (*Vulpes vulpes*), eight marbled polecats (*Vormela peregusna*), six Asian badgers (*Meles leucurus*), three Eurasian lynxes (*Lynx lynx*), five long-eared hedgehogs (*Hemiechinus auritus*), 121 lizards, 228 birds, three sika deer (*Cervus nippon*), one Bactrian camel (*Camelus bactrianus*), one wolf (*Canis lupus*), two Serengeti lions (*Panthera leo*), one tiger (*Panthera tigris*), one brown bear (*Ursus arctos*), one Asiatic black bear (*Ursus thibetanus*), one rhesus monkeys (*Macaca mulatta*), three emus (*Dromaius novaehollandiae*) and one peafowl (*Pavo cristatus*)(Additional file 1: Table S1; Additional file 2: Table S2; Fig. 1). The species of sampled hosts were identified by key morphological characteristics [10, 11] and by genetic markers including the 910-bp *16S rRNA* fragment for birds [12], the 924-bp displacement loop (D-loop) mitochondrial DNA (mtDNA) for bird feces [13], the 650-bp cytochrome* c* oxidase subunit I (*COX1*) for reptiles [14] and a 1242-bp cytochrome B (*cytb*) sequence for rodents (Additional file 3) [15].

### DNA extraction

Genomic DNA from each intestinal sample was extracted using the TIANamp Genomic DNA Kit (TIANGEN, Beijing, China) following the manufacturer’s instructions. Genomic DNA from each fecal sample was extracted using the EasyPure Stool Genomic DNA Kit (TRANS, Beijing, China). The DNA quantity was assessed on a NanoDrop 2000 spectrophotometer (Thermo Fisher Scientific, Waltham, MA, USA). Samples with a DNA concentration of at least 30 ng/μl could be used to detect pathogens.

### Molecular detection of *Enterocytozoon bieneusi*

*Enterocytozoon bieneusi* was identified and genotyped using a nested PCR protocol with both forward and reverse primers for the sequence, targeting an approximately 390-bp nucleotide fragment in the *ITS* region [16]. The PCR products were purified using the TIANgel Midi Purification Kit (TIANGEN) and sequenced.

### Nucleotide sequencing and analysis

The obtained sequences were edited and compared to sequences from GenBank using the BLASTN program (http://www.ncbi.nlm.nih.gov/BLAST/). Fifty sequences that served to identify host species were deposited in the GenBank database (*16S rRNA*: PQ394778-PQ394785, PQ451723-PQ451727 and PQ459365; *COX*1: PQ451734-PQ451736; D-loop mtDNA: PQ373942-PQ373943, PQ393132-PQ393138 and PQ474771; *cytb*: PQ450150-PQ450162, PQ450164-PQ450166, PQ474772-PQ474773, OR548119 and PQ581939). In addition, 23 sequences of *E. bieneusi* from this study were also deposited in GenBank, under accession numbers PP489440-PP489449, PP601260, PP959050-PP959059 and PQ489475-PQ489476.

### Phylogenetic analyses

A neighboring-joining (NJ) phylogenetic tree was constructed for *E. bieneusi* using the maximum composite likelihood model with MEGA 7.0 software. Bootstrap values were obtained with 1000 replicates.

### Statistical analyses

Prevalence data were compared by Fisher’s exact test, and differences were regarded significant when *p* < 0.05.

## Results

### Identification of wild animal species

In this study, 1372 samples were collected from 68 species of animals, including 34 mammalian, six reptilian and 28 avian species (Table 1).

**Table 1:** Prevalence and distribution of *Enterocytozoon bieneusi* genotypes in wild animals in northwest China

Animal	Number of samples obtained and examined	Number of positive samples (%)	*Enterocytozoon bieneusi* genotype(s) (no. of positive samples)	Genogroup(s) (no. of positive samples)^a^
*Class Mammalia*	1019	123 (12.1)		
Rodentia	610	83 (13.6)	PL14 (*n* = 42), EbpD (*n* = 13), XJHT4 (*n* = 6), D (*n* = 3), horse1 (*n* = 18), CHG7 (*n* = 1)	1 (*n* = 35), 12 (*n* = 42), 14 (*n* = 6)
Lagomorpha	82	1 (1.2)	BEB6 (n = 1)	2 (*n* = 1)
Soricomorpha	2	0		
Perissodactyla	273	30 (11.0)	CHG7 (*n* = 29), horse1 (*n* = 1)	1 (*n* = 30)
Even-toed ungulate	7	0		
Carnivora	39	9 (23.1)	NCF2 (*n* = 4), NCF6 (*n* = 3), D (*n* = 2)	1 (*n* = 9)
Erinaceomorpha	5	0		
Primates	1	0		
*Class Reptilia*	131	0		
*Class Aves*	232	10 (4.3)	MWC-d1 (*n* = 2), CHG7 (*n* = 3), E (*n* = 1), SN45 (*n* = 1), horse1 (*n* = 3)	1 (*n* = 10)
Anseriformes	48	2 (4.2)	MWC-d1 (*n* = 2)	1 (*n* = 2)
Lariformes	98	3 (3.1)	CHG7 (*n* = 3)	1 (*n* = 3)
Accipitriformes	20	2 (10.0)	horse1 (*n* = 1), E (*n* = 1)	1 (*n* = 2)
Passeriformes	39	2 (5.1)	SN45 (*n* = 1), horse1 (*n* = 1)	1 (*n* = 2)
Cuculiformes	5	0		
Columbiformes	8	0		
Bucerotiformes	3	0		
Strigiformes	3	1 (33.4)	horse1 (*n* = 1)	1 (*n* = 1)
Coraciiformes	1	0		
Caprimulgiformes	2	0		
Struthioniformes	3	0		
Galliformes	1	0		
Gruiformes	1	0		
*Total*	1372	133 (9.7)	PL14 (*n* = 42), CHG7 (*n* = 33), horse1 (*n* = 22), EbpD (*n* = 13), XJHT4 (*n* = 6), D (*n* = 5), NCF2 (*n* = 4), NCF6 (*n* = 3), MWC_d1 (*n* = 2), BEB6 (*n* = 1), E (*n* = 1), SN45 (*n* = 1)	1 (*n* = 84), 2 (*n* = 1), 12 (*n* = 42), 14 (*n* = 6)

^a^In the southwestern and central regions of China, *Enterocytozoon bieneusi* genotypes have been assigned to 14 major genetic groups, denoted as Group 1 to 14

### Prevalence of *E. bieneusi*

We detected *E. bieneusi* in 9.7% (133/1372) of samples, including 11.9% (121/1019) of samples from mammals, 4.3% (10/232) of samples from birds and none of the samples (0/121) from reptiles (Table 1). Among the samples from mammals, the highest rate of infection was detected in carnivores (23.1%) and rodents (13.6%). Within the same genus of rodents, the prevalence was significantly higher in *Meriones tamariscinus* than in *Meriones libycus*, and in *Spermophilus erythrogenys* compared to *Spermophilus undulatus* (*p* < 0.0001) (Additional file 1: Table 1).

### Genotypes of* E. bieneusi*

**Fig. 2:**
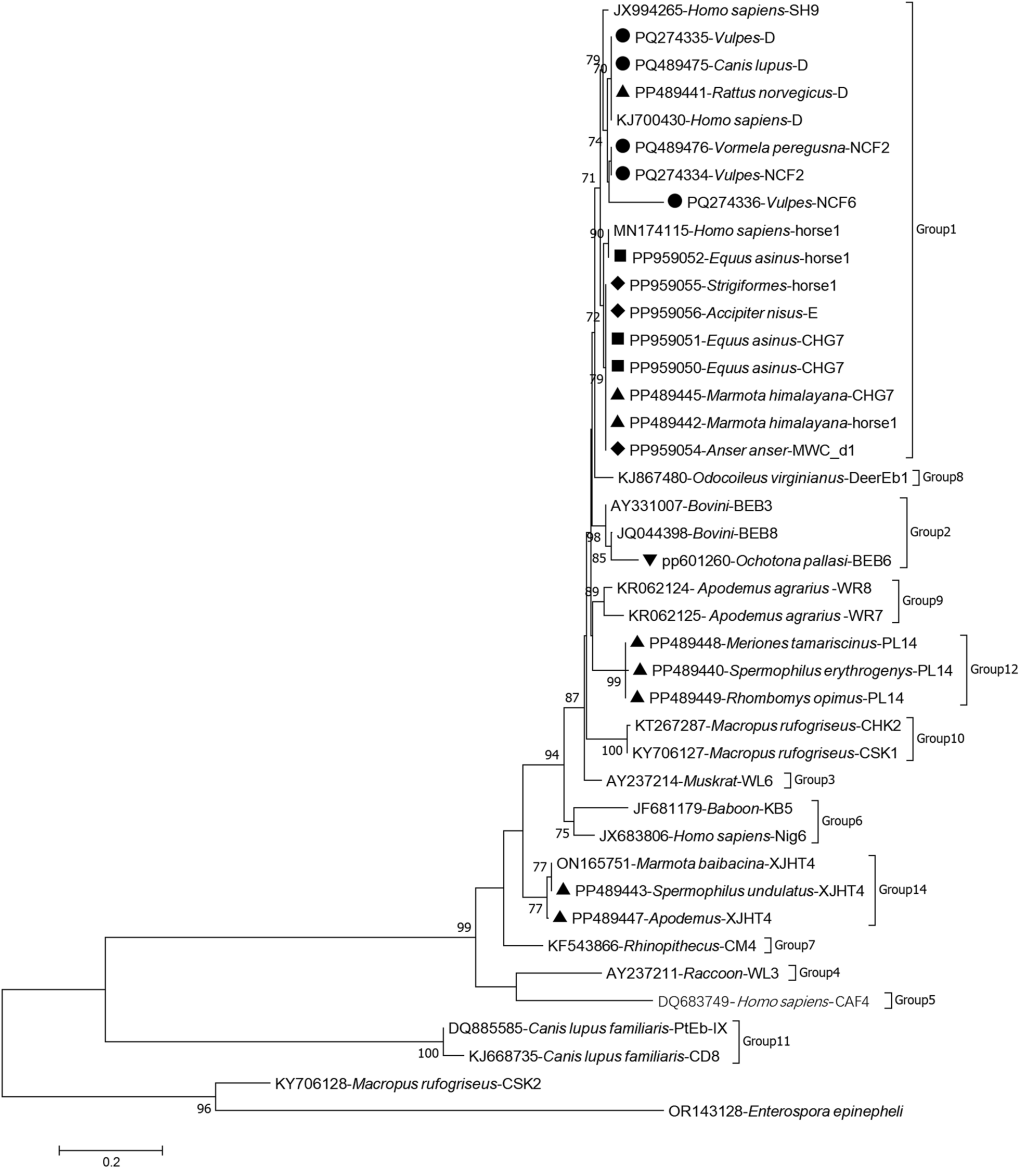
Phylogenetic relationships of *Enterocytozoon bieneusi* genotypes identified in the present study and other known genotypes deposited in GenBank, inferred from *ITS* sequences analyzed with the neighboring-joining method using the maximum composite likelihood model, with 1000 replicates. Each sequence is shown according to its accession number, host origin and genotype designation. Rodents (black triangles), *Ochotona pallasi* (Mongolian pika; black inverted triangles), *Equus asinus* (wild asses; black squares), carnivores (black circles) and birds (black diamonds) are indicated

Based on the *ITS* region, a total of 12 genotypes of *E*. *bieneusi* were identified, including BEB6 (synonyms: CHS4, CHC9, CHHLJS1, CHS18, JSS1 and SH5), CHG7, D (synonyms: WL8, Peru9, PigEBITS9, PtEb VI and CEbC), E (synonyms: EbpC, WL13, Peru4 and WL17), EbpD, horse1, MWC_d1 (synonym: BJED-V), NCF2, NCF6, PL14, SN45 and XJHT4 (Fig. 2). All *ITS* sequences showed 98.0–100.0% similarity to the following GenBank reference sequences: MW429409 for genotype BEB6; KP262358 for genotype CHG7; MT895457 for genotype D; KJ700426 for genotype E; KJ728797 for genotype EbpD; MW429428 for genotype horse1; MK121776 for genotype MWC_d1; MG976814 for genotype NCF2; PQ165135 for genotype NCF6; MZ400637 for genotype PL14; MN378369 for genotype SN45; and ON165751 for genotype XJHT4.

Among these *E. bieneusi* genotypes, PL14 (31.6%, 42/133) was the most prevalent, followed by the CHG7 (24.8%, 33/133), horse1 (16.5%, 22/133), EbpD (9.8%, 13/133), XJHT4 (4.5%, 6/133), D (3.8%, 5/133), NCF2 (3.0%, 4/133), NCF6 (2.3%, 3/133), MWC_d1 (1.5%, 2/133), BEB6 (0.8%, 1/133), E (0.8%, 1/133) and SN45 genotypes (0.8%, 1/133). The distribution of these among different host species is shown in Table 1.

We observed a varied distribution pattern of the *E. bieneusi* genotypes among different animal species. Genotypes EbpD, PL14 and XJHT4 were only found in rodent species that were positive for the pathogen. Genotypes E, MWC_d1 and SN45 were only found in bird species, and genotypes NCF2 and NCF6 were only found in carnivores*.*

### Phylogenetic relationships of *E. bieneusi* genotypes

Genotypes identified in this study represented four major phylogenetic groups, namely Groups 1, 2, 12 and 14. Genotypes CHG7, D, E, EbpD, horse1, MWC_d1, NCF2, NCF6 and SN45 clustered in Group 1. At the same time, genotypes BEB6, PL14 and XJHT4 belonged to Groups 2, 12 and 14, respectively (Fig. 2).

## Discussion

In this study, most of our genotypes of *E*. *bieneusi* fall into Group 1, including genotypes CHG7, D, E, EbpD, horse1, MWC_d1, NCF2, NCF6 and SN45. Group 1 is the largest genotype group, comprising 314 genotypes among which genotypes D, E and Type IV are widely spread across host species and geographic ranges and, importantly, they are also frequently found in human beings. According to a previous multi-locus sequence typing (MLST) analysis, Group 1 is further divided into eight subgroups, from subgroup 1a to 1h [17].

Wildlife is a potential source of human infections of *E*. *bieneusi* in central and southern China. In this study, we detected *E*. *bieneusi* in 9.4% (103/1099) of the samples from wild animals. This prevalence is lower than reported than that reported other studies; for example, *E*. *bieneusi* prevalence in animals from Zhengzhou Zoo and Chengdu Zoo was reported to be the same, namely 15.8% (32/203) [18] and 15.8% (43/272) [19], respectively, while the prevalence of *E. bieneusi* from Shanghai Zoo and Zhejiang Zoo was 11.5% (21/182) [20]. The difference in *E*. *bieneusi* prevalence between our results and those of previous studies may be due to differences in geographical location, in habitats and/or in host species.

Rodents, especially wild-living species, can act as reservoirs for numerous pathogens, and some of these pathogens (as also exemplified by *E. bieneusi*) are zoonotic [6, 21]. There are over 2000 species of rodents worldwide. Previously, 60 genotypes of *E. bieneusi*, among them 18 zoonotic ones, have been confirmed in rodents in different parts of Eurasia, including China [2, 8, 17, 22–26]. In addition, genotype PL14, representing phylogenetic group 12, has been occasionally identified in farmed masked palm civet (*Paguma larvata*) in Yunnan Province, southwestern China [27]. In the present study, we identified genotype PL14 for the first time in rodents, including the red-cheeked ground squirrel (*Spermophilus erythrogenys*), Tamarisk gerbils (*Meriones tamariscinus*) and the great gerbil (*Rhombomys opimus*). On the other hand, genotype XJHT4 was previously reported in samples from gray marmots (*Marmota baibacina*) [28]. Our results indicate broadening of its host spectrum, with genotype XJHT4 also detected in the long-tailed ground squirrel (*Spermophilus undulatus*) and pygmy wood mouse (*Apodemus uralensis*)*.* Genotype D was the most frequently detected variant in rodents in Poland and the border region of Czech Republic-Germany [21]. In contrast, PL14 was the most dominant genotype in rodents in the present study, with other pathogenic genotypes, including CHG7, D, EbpD, horse1 and XJHT4, also shown to be present in local rodents in northern China.

Pikas (*Ochotonidae*) are mainly distributed in Central Asia, but their range also includes northeastern Asia, western North America and Europe [29]. There are only 30 species of pikas, of which 24 species occur in China [30]. *Enterocytozoon bieneusi* genotypes CHN14 and CHS17 have been observed previously in plateau pikas (*Ochotona curzoniae*) [23]. In the present study, we found genotype BEB6, a member of phylogenetic Group 2, for the first time in Mongolian pikas (*Ochotona pallasi*) collected in Beitashan Mountain, XUAR. Interestingly, BEB6 was previously identified in Tibetan sheep and yaks [31]. Beitashan Mountain, covering 3848 km^2^ along the China-Mongolia border, has a fauna rich in wild animal species that inhabit alpine meadows, such as pikas, yaks and sand gazelles (*Gazella subgutturosa*). Therefore, in the future, the epidemiological significance of transmission routes involving these mammals should be further evaluated.

Genotype D of *E. bieneusi* is widely distributed in animal and human patients [1, 5, 22], and genotypes NCF2 and NCF6 may infect a broad range of carnivores, such as raccoons (*Procyon lotor*), red foxes (*Vulpes vulpes*), European badgers (*Meles meles*) [32] and arctic foxes (*Vulpes lagopus*) [32, 33]. In the present study study, several new host associations were revealed, including genotype NCF2 in marbled polecats (*Vormela peregusna*), genotypes NCF6 in red foxes (*Vulpes vulpes*) and genotype D in the gray wolf (*Canis lupus*). These findings extend the host ranges of genotypes D, NCF2 and NCF6. Interestingly, genotype NCF2 was detected in both marbled polecats and red foxes, and genotype D in rodents, red fox and gray wolf. These data suggest that red foxes play a pivotal role in pathogen spillover via the predator–prey relationship, and that *E. bieneusi* can be transmitted via the “rodent*-*red fox-gray wolf” or “marbled polecat-red fox” food chain (Additional file 4).

*Enterocytozoon bieneusi* genotype horse1 is widely found in equines, but genotype CHG7 was previously detected only in pigs and sheep [34, 35]. In the present study, genotypes horse1 and CHG7 were both detected in donkeys imported from Kyrgyzstan. Similarly, genotype SN45 was previously found in wild Himalayan marmots (*Marmota himalayana*) and Alashan ground squirrels (*Spermophilus alashanicus*) [37], while genotype MWC_d1 was only shown to be present in wild ungulate species [37, 38]. We report here for the first time the detection of genotypes MWC_d1 and SN45 in migratory birds [such as the greylag goose (*Anser anser*) and northern wheatear (*Oenanthe oenanthe*)]. There are three international flyways in XUAR: (i) the West Asia-Middle East-East Africa flyway; (ii) the Siberia-Central Asia-South Asia flyway; and (iii) the Arctic tundra-Asia-Australia flyway. Multiple genotypes of *E. bieneusi* were detected in imported donkeys and migratory birds in our study, and some of these were shared between these two host types, indicating that the host range of *E. bieneusi* genotypes is extended due to pathogen spillover, and that the risks of transmission of *E. bieneusi* across the borders are high in XUAR.

There is an earlier report of a pathogen spillover-related outbreak between migratory birds and resident birds, as in the case of genotype Peru6 transmission between red-crowned crane (*Grus japonensis*) and pigeons (*Columba livia*) [40]. In the present study, genotype CHG7 of *E. bieneusi* was detected in both wild-living resident birds (e.g. red-naped ibis) and migratory birds [e.g., Pallas’s gull (*Larus ichthyaetus*) off the coast of Ganjam and lesser black-backed gull (*Larus fuscus*)], indicating that some *E. bieneusi* genotypes circulate between resident birds and migratory birds in northwestern China.

No published reports are available on *E. bieneusi* infection in lizards. In this study, *E. bieneusi* was not detected in lizard samples, possibly due to pathogen specificity or rare infection in lizards. At the same time, *E. bieneusi* genotype horse1 was widely distributed among rodents (e.g. Himalayan marmots) and resident birds [e.g. long-eared owls (*Asio otus*), jungle nightjars (*Caprimulgus indicus*) and Hume’s groundpeckers (*Pseudopodoces humilis*)] in their habitat-overlap region, suggesting that genotype horse1 is transmitted via the fecal–oral route (Additional file 5).

Despite the significance of the findings of this study, there are some limitations because the species diversity in regional wildlife is only partly represented. Therefore, it is probably that numerous examples of predator–prey relationships that are important in the epidemiology of *E. bieneusi* remain to be explored.

## Conclusions

A total of 1372 samples, collected from 34 mammalian, six reptilian and 28 avian species, were examined in this study. Twelve genotypes of *E. bieneusi*, including BEB6, CHG7, D, E, EbpD, horse1, MWC_d1, NCF2, NCF6, PL14, SN45 and XJHT4, were identified in the samples collected from XUAR, IMAR and Kyrgyzstan. These genotypes represent four phylogenetic groups, namely Group 1, Group 2, Group 12 and Group 14. These findings indicate that red foxes play a vital role in pathogen spillover via the predator–prey relationship; that the risk of cross-border transmission of *E. bieneusi* is high in XUAR owing to imported livestock and migratory birds; and that the genotype horse1 circulates between marmots and resident birds in habitat-overlap regions due to fecal–oral transmission.

## Supplementary Information

Additional file 1: Table S1. Prevalence and distribution of *Enterocytozoon bieneusi* genotypes in wild animals in Northwest China.

Additional file 2: Table S2. Animal species used as sources of samples in the study.

**Additional file 3:** Characteristics of amplified fragments and corresponding primer sequences.

**Additional file 4:** In this study, transmission routes involving the predator-prey relationship between hosts of genotype D and genotype NCF2 were investigated.

**Additional file 5:** Genotype horse1 was detected in Himalayan marmots, owls, jungle nightjars and Hume’s groundpeckers, revealing habitat overlap in these animals.

## Data Availability

No datasets were generated or analysed during the current study.

## Abbreviations

*16S rRNA*: 16S Ribosomal RNA
*COX1*: Cytochrome* c* oxidase subunit I
*Cyt**b*: Cytochrome B
D-loop: Displacement loop region
