# Supplementary material for: Genetic diversity of Enterocytozoon bieneusi in 1099 wild animals and 273 imported pastured donkeys in northern China
Source: Parasit Vectors. 2025 Mar 13;18:105. doi: 10.1186/s13071-025-06739-6 (PMC11905730; doi:10.1186/s13071-025-06739-6)
Supplement: Supplementary file 1 — Additional file 1: Table S1. Prevalence and distribution of Enterocytozoon bieneusi genotypes in wild animals in Northwest China. [file 13071_2025_6739_MOESM1_ESM.pdf]

Addition file 1. Table S1.

Prevalence and distribution of *E. bieneusi* genotypes in wild animals in Northwest China.

Animal	No. examined	No. positive (%)	Genotype(s) (No. of positive samples)	Genogroup(s) (No. of positive samples)
Mammalia	1019	123 (12.1)		
Rodentia	610	83 (13.6)	PL14 (n=42)，EbpD (n=13)，XJHT4 (n=6)，D (n=3)，horse1 (n=18)，CHG7 (n=1)	1 (n=35), 12 (n=42), 14 (n=6)
Marmota himalayana	95	19 (20.0)	horse1 (n=18), CHG7 (n=1)	1 (n=18), 12 (n=1)
Marmota baibacina	8	0		
Rhombomys opimus	152	16 (10.5)	EbpD (n=13)， PL14 (n=3)	1 (n=13),12 (n=3)
Meriones tamariscinus	14	7 (50.0)	PL14 (n=7)	12 (n=7)
Meriones libycus	42	0		
Spermophilus undulatus	109	5 (4.6)	XJHT4 (n=5)	14 (n=5)
Spermophilus erythrogenys	115	32 (27.8)	PL14 (n=32)	12 (n=32)
Spermophilus alashanicus	11	0		
Rattus norvegicus	18	3 (16.7)	D (n=3)	1 (n=3)
Apodemus uralensis	1	1 (100.0)	XJHT4 (n=1)	14 (n=1)
Mus musculus	7	0		
Microtus arvalis	20	0		
Ellobius talpinus	13	0		
Dipus sagitta	1	0		
Allactaga sibirica	1	0		
Ondatra zibethicus	5	0		
Lagomorpha				
Ochotona pallasi	81	1 (1.2)	BEB6 (n=1)	2 (n=1)
Lepus yarkandensis	1	0		
Soricomorpha				

Sorex araneus	2	0		
Perissodactyla				
Equus asinus	273	30 (11.0)	CHG7 (n=29), horse1 (n=1)	1 (n=30)
Even-toed ungulate				
Procapra przewalskii	3	0		
Cervidae	3	0		
Camelus bactrianus	1	0		
Carnivora				
Vulpes vulpes	16	7 (43.8)	NCF2 (n=3), NCF6 (n=3), D (n=1)	1 (n=7)
Vormela peregusna	8	1 (12.5)	NCF2 (n=1)	1 (n=1)
Meles meles	6	0		
Lynx lynx	3	0		
Canis lupus	1	1 (100.0)	D (n=1)	1 (n=1)
Panthera leo	2	0		
Ursus arctos	1	0		
Ursus thibetanus	1	0		
Panthera tigris	1	0		
Erinaceomorpha				
Erinaceus	5	0		
Primates				
Cercopithecidae	1	0		
Reptilia	131	0		
Phrynocephalus				
Eremias velox	40	0		
Eremias				
Phrynocephalus versicolor	20	0		
Phrynocephalus guttatus	24	0		
Laudakia				

Paralaudakia lehmanni	11	0		
Paralaudakia caucasia	16	0		
Paralaudakia microlepis	10	0		
Aves	232	10 (4.3)	MWC-d1 (n=2), CHG7 (n=3), E (n=1), SN45 (n=1), horse1 (n=3)	1 (n=10)
Anseriformes				
Anser anser	48	2 (4.2)	MWC_d1 (n=2)	1 (n=2)
Lariformes				
Larus ichthyaetus	20	1 (5.0)	CHG7 (n=1)	1 (n=1)
Larus fuscus	16	1 (6.3)	CHG7 (n=1)	1 (n=1)
Larus argentatus	17	0		
Larus armenicus	5	0		
Larus cachinnans	14	0		
Larus hemprichii	16	0		
Charadrius dubius	6	0		
Ciconia nigra	2	1 (50.0)	CHG7 (n=1)	1 (n=1)
Larus argentatus	2	0		
Accipitriformes				
Caprimulgus indicus	12	1 (8.3)	horse1 (n=1)	1 (n=1)
Accipiter nisus	8	1 (12.5)	E (n=1)	1 (n=1)
Passeriformes				
Passer montanus	10	0		
Alauda arvensis	4	0		
Oenanthe oenanthe	4	1 (25.0)	SN45 (n=1)	1 (n=1)
Turdus merula	12	0		
Pseudopodoces humilis	3	1 (33.4)	horse1 (n=1)	1 (n=1)
Sturnus vulgaris	2	0		
Melanocy phamongollca	4	0		

Cuculiformes				
Cuculus canorus	5	0		
Columbiformes				
Streptopelia decaocto	7	0		
Streptopelia orientalis	1	0		
Bucerotiformes				
Upupa epops	3	0		
Strigiformes				
Strigiformes	3	1 (33.4)	horse1 (n=1)	1 (n=1)
Coraciiformes				
Coracias garrulus	1	0		
Caprimulgiformes				
Caprimulgus europaeus	2	0		
Struthioniformes				
Struthio camelus	3	0		
Galliformes				
Pavo muticus	1	0		
Gruiformes				
Fulica atra	1	0		
**Total**	1372	133 (9.7)	PL14 (n=42), CHG7 (n=33), horse1 (n=22), EbpD (n=13), XJHT4 (n=6), D (n=5), NCF2 (n=4), NCF6 (n=3), MWC_d1 (n=2), BEB6 (n=1), E (n=1), SN45 (n=1)	1 (n=84), 2 (n=1), 12 (n=42), 14 (n=6)
