# Supplementary material for: Genetic diversity of Enterocytozoon bieneusi in 1099 wild animals and 273 imported pastured donkeys in northern China
Source: Parasit Vectors. 2025 Mar 13;18:105. doi: 10.1186/s13071-025-06739-6 (PMC11905730; doi:10.1186/s13071-025-06739-6)
Supplement: Supplementary file 2 — Additional file 2: Table S2. Animal species used as sources of samples in the study. [file 13071_2025_6739_MOESM2_ESM.pdf]

Additional file 2. Table S2.

Animal species used as sources of samples in the study.

**Animal**	**No. examined**	**sample type**	**collection time**	**collection position**	**species identification**	**permit**
Mammalia	1019	small intestine				
Rodentia	610	small intestine			GenBank Number NO.	
Marmota himalayana	95	small intestine	2021.4.10-2023.6.16	Hotan Prefecture in XUAR	PQ581939	Approval No. A2015- 063- 01
Marmota baibacina	8	small intestine	2021.4.10-2023.6.16	Hotan Prefecture in XUAR	PQ450150	Approval No. A2015- 063- 01
Rhombomys opimus	152	small intestine	2020.5.25-2023.8.25	Ganqimaodu town in IMAR and XUAR	PQ450151	Approval No. A2018-143-01
Meriones tamariscinus	14	small intestine	2023.2.19	149 Regiment of XUAR	PQ450156	Approval No. A2022-029-01
Meriones libycus	42	small intestine	2023.8.13	Liugou reservoir in XUAR	PQ450155	Approval No. A2022-029-01
Spermophilus undulatus	109	small intestine	2022.8.2-2023.6.19	Jinghe County, Wusu County, Altay Prefecture and Korla in XUAR	PQ474772	Approval No. A2022-029-01
Spermophilus erythrogenys	115	small intestine	2022.7.25-2023.4.27	Toli County and Mongolian Autonomous County of Hoboksar in XUAR	PQ450152	Approval No. A2022-029-01
Spermophilus alashanicus	11	small intestine	2023.4.27	Toli County in XUAR	PQ450154	Approval No. A2022-029-01

Rattus norvegicus	18	small intestine	2023.7.19-20	Shihezi City in XUAR	PQ450157	Approval No. A2022-029-01
Apodemus	1	small intestine	2023.10.4	Alataw Pass in XUAR	PQ450166	Approval No. A2022-029-01
Mus musculus	7	small intestine	2023.10.15	Alataw Pass inXUAR	PQ450165	Approval No. A2022-029-01
Microtus arvalis	20	small intestine	2015.10.3	Tangbula in XUAR	PQ450161	Approval No. AECSU2013-18
Ellobius talpinus	13	small intestine	2019.7.23	Wujiaqu in XUAR	PQ450162	Approval No. AECSU2013-18
Dipus sagitta	1	small intestine	2023.10.4	Manasi County in XUAR	PQ450159	Approval No. A2022-029-01
Allactaga sibirica	1	small intestine	2021.10-2022.5	Pishan farm in XUAR	PQ450158	Approval No. A2022-029-01
Ondatra zibethicus	5	small intestine	2021.10-2022.5	Tumushuke City in XUAR	PQ474773	Approval No. A2022-029-01
Lagomorpha						
Ochotona pallasi	81	small intestine	2021-2023	Beitashan Mountain in XUAR	OR548119.1	Approval no. A2021-053-01
Lepus yarkandensis	1	small intestine	2023.9.22	Tumushuke City in XUAR	PQ450164	Approval No. A2022-029-01
Soricomorpha						
Sorex araneus	2	small intestine	2023.3.21	149 Regiment of XUAR	PQ450160	Approval no. A2021-053-01
Perissodactyla						
Equus asinus	273	small intestine	2023.11-2024.5.29	Slaughterhouse in Uqia County in XUAR	morphological identification	Approval No. A2022-029-01
Artiodactyla						
Procapra przewalskii	3	small intestine	24.1.16-24.4.24	Liugou reservoir in XUAR	morphological	Approval No. A2022-029-01

					identification	
Cervidae	3	faeces	24.8.30	Shihezi Zoo	morphological identification	Approval No. A2022-029-01
Camelus bactrianus	1	faeces	2024.10.17	Shihezi Zoo	morphological identification	Approval No. A2022-029-01
Carnivora						
Vulpes Vulpes	16	small intestine	2015.10-2024.4.24	Nilka County in XUAR	morphological identification	Approval No. AECSUKJ2015−01
Vormela peregusna	8	small intestine	2014.5-2024.10.1	Ebinur Lake and Karamay in XUAR	morphological identification	Approval No. AECSU2014-03
Meles meles	6	small intestine	2018.3	Nilka County in XUAR	morphological identification	Approval No. A2018-143-01
Lynx lynx	3	small intestine	2019.09.26	the West Junggar Mountains in XUAR	morphological identification	Approval numbers 2015-063-01 and A2018-144- 01.
Canis lupus	1	faeces	2024.10.17	Shihezi Zoo	morphological identification	Approval No. A2022-029-01
Panthera leo	2	faeces	2024.10.17	Shihezi Zoo	morphological identification	Approval No. A2022-029-01
Ursus arctos	1	faeces	2024.10.17	Shihezi Zoo	morphological	Approval No. A2022-029-01

					identification	
Ursus thibetanus	1	faeces	2024.10.17	Shihezi Zoo	morphological identification	Approval No. A2022-029-01
Panthera tigris	1	faeces	2024.10.17	Shihezi Zoo	morphological identification	Approval No. A2022-029-01
Erinaceomorpha						
Erinaceus	5	small intestine	2023.8.26	Ganqimaodu town in IMAR	morphological identification	Approval No. A2022-029-01
Primates						
Cercopithecidae	1	small intestine	2024.8.18	Shihezi Zoo	morphological identification	Approval No. A2022-029-01
Reptilia	121	small intestine			GenBank Number NO.	
Eremias						
Eremias velox	40	small intestine	2022.5.24-2023.7.22	Chamalle Forest Farm and Wusu city in XUAR	PQ451734	Approval No. A2022-029-01
Phrynocephalus						
Phrynocephalus versicolor	20	small intestine	2023.7.22	Wusu city in XUAR	PQ451735	Approval No. A2022-029-01
Phrynocephalus guttatus	24	small intestine	2023.7.22	Wusu city in XUAR	PQ451736	Approval No. A2022-029-01
Laudakia						

Paralaudakia lehmanni	11	small intestine	2024.7.2	Beitashan Mountain in XUAR	PQ459404	Approval No. A2022-029-01
Paralaudakia caucasia	16	small intestine	2024.7.2	Beitashan Mountain in XUAR	PQ459403	Approval No. A2022-029-01
Paralaudakia microlepis	10	small intestine	2024.7.2	Beitashan Mountain in XUAR	PQ459402	Approval No. A2022-029-01
Aves	232					
Anseriformes						
Anser anser	48	faeces	24.4.10	Moguhu reservoir in XUAR	PQ393132	Approval No. A2022-029-01
Charadriiformes						
Larus ichthyaetus	20	faeces	24.4.23	Liugou reservoir in XUAR	PQ393136	Approval No. A2022-029-01
Larus fuscus	23	faeces	24.4.23	Liugou reservoir in XUAR	PQ393133	Approval No. A2022-029-01
Larus armenicus	5	faeces	24.4.23	Liugou reservoir in XUAR	PQ393138	Approval No. A2022-029-01
Larus cachinnans	14	faeces	24.4.23	Liugou reservoir in XUAR	PQ393134	Approval No. A2022-029-01
Larus hemprichii	16	faeces	24.4.23	Liugou reservoir in XUAR	PQ393135	Approval No. A2022-029-01
Charadrius dubius	6	faeces	24.4.23	Liugou reservoir in XUAR	PQ393137	Approval No. A2022-029-01
Larus argentatus	2	faeces	24.4.23	Liugou reservoir in XUAR	PQ474771	Approval No. A2022-029-01
Ciconiiformes						
Ciconia nigra	2	faeces	24.4.23	Liugou reservoir in XUAR	PQ459365	Approval No. A2022-029-01

Accipitriformes						
Accipiter nisus	8	small intestine	24.5.30	Shihezi Airport	PQ394781	Approval No. A2022-029-01
Passeriformes						
Lanius collurio	10	small intestine	24.5.30 - 24.6.22	Shihezi Airport	PQ451724	Approval No. A2022-029-01
Alauda razae	4	small intestine	24.6.15	Shihezi Airport	PQ451725	Approval No. A2022-029-01
Oenanthe oenanthe	4	small intestine	24.5.30	Shihezi Airport	PQ394779	Approval No. A2022-029-01
Turdus merula	10	small intestine	24.5.30 - 24.6.22	Shihezi Airport	PQ394784	Approval No. A2022-029-01
Pseudopodoces humilis	3	small intestine	24.5.30	Shihezi Airport	PQ394778	Approval No. A2022-029-01
Sturnus vulgaris	2	small intestine	24.6.12	Shihezi Airport	PQ394785	Approval No. A2022-029-01
Calandrella cinerea	4	small intestine	24.6.22	Shihezi Airport	PQ451723	Approval No. A2022-029-01
Hirundo rustica	2	small intestine	24.6.22	Shihezi Airport	PQ451727	Approval No. A2022-029-01
Cuculiformes						
Cuculus canorus	5	small intestine	24.5.30 - 24.6.22	Shihezi Airport	PQ394783	Approval No. A2022-029-01
Columbiformes						
Streptopelia orientalis	8	small intestine	24.6.12	Shihezi Airport	PQ394782	Approval No. A2022-029-01
Bucerotiformes						
Upupa epops	3	small intestine	24.6.22	Shihezi Airport	PQ451726	Approval No. A2022-029-01
Strigiformes						

Strigiformes	3	small intestine	24.5.30	Shihezi Airport	morphological identification	Approval No. A2022-029-01
Coraciiformes						
Coracias garrulus	1	small intestine	24.6.22	Shihezi Airport	morphological identification	Approval No. A2022-029-01
Caprimulgiformes						
Caprimulgus europaeus	2	small intestine	24.6.22	Shihezi Airport	morphological identification	Approval No. A2022-029-01
Caprimulgus indicus	12	small intestine	24.5.30	Shihezi Airport	PQ394780	Approval No. A2022-029-01
Struthioniformes						
Struthio camelus	3	small intestine	24.5.7	Shihezi Zoo	morphological identification	Approval No. A2022-029-01
Galliformes						
Pavo muticus	1	small intestine	24.5.7	Shihezi Zoo	morphological identification	Approval No. A2022-029-01
Gruiformes						
Fulica atra	1	small intestine	24.4.24	Liugou reservoir in XUAR	morphological identification	Approval No. A2022-029-01
Total	1372					

Comment：Although a grey wolf in this study came from the zoo, it was adult wolf and lived in the wild until it was 3 years old, and was transferred to the zoo after 3 years old, so it may be susceptible to wild predation disease.
