# Supplementary material for: Genetic diversity of Enterocytozoon bieneusi in 1099 wild animals and 273 imported pastured donkeys in northern China
Source: Parasit Vectors. 2025 Mar 13;18:105. doi: 10.1186/s13071-025-06739-6 (PMC11905730; doi:10.1186/s13071-025-06739-6)
Supplement: Supplementary file 3 — Additional file 3: Characteristics of amplified fragments and corresponding primer sequences. [file 13071_2025_6739_MOESM3_ESM.pdf]

Additional file 3.

Characteristics of amplified fragments and corresponding primer sequences.

The PCR equipment was a Mastercycler X50s, Eppendorf, Germany.

1. **PCR amplification for identification of the rat species based on the cytochrome b *(cytb)* gene**

The PCR amplifications were performed in a 25-μL reaction volume. The reaction mixture contained 0.75 μmol/L of *Taq* polymerase (2×Rapid *Taq* Master Mix, Vazyme,China).

For PCR primer1, the PCR cycle had the following parameters: 95°C for 5 min, 35 cycles at 94°C for 30s, 55°C for 60s, 72°C for 60s, and a final step at 72°C for 10 min.

For PCR primer2,the PCR cycle had the following parameters: 94°C for 3 min, 35 cycles at 94°C for 35s, 53°C for 35s, 72°C for 80s, and a final step at 72°C for 10 min.

2. **PCR amplification for identification of bird species based on the 16S rRNA gene.**

The PCR amplifications were performed in a 25-μL reaction volume. The reaction mixture contained 0.75 μmol/L of *Taq* polymerase (2×Rapid *Taq* Master Mix, Vazyme,China).

The PCR cycle had the following parameters: 95°C for 5 min, 35 cycles at 95°C for 30s, 55°C for 40s, 72°C for 60s, and a final step at 72°C for 10 min.

3. **PCR amplification for identification of bird species based on the D-loop-mtDNA .**

The PCR amplifications were performed in a 25-μL reaction volume. The reaction mixture contained 0.75 μmol/L of *Taq* polymerase (2×Rapid *Taq* Master Mix, Vazyme,China).

The PCR cycle had the following parameters: 94°C for 3 min, 35 cycles at 94°C for 30s, 59°C for 30 s, 72°C for 30s, and a final step at 72°C for 10 min.

4. **PCR amplification for identification of the lizard species based on the *COX1* gene.**

The PCR amplifications were performed in a 25-μL reaction volume. The reaction mixture contained 0.75 μmol/L of *Taq* polymerase (2×Rapid *Taq* Master Mix, Vazyme,China).

The PCR cycle had the following parameters: 94°C for 3 min, 40 cycles at 94°C for 30s, 50°C for 30s, 72°C for 30s, and a final step at 72°C for 10 min.

5. PCR amplification to detect *E. bieneusi* based on the *ITS* gene

The PCR amplifications were performed in a 25-μL reaction volume. The reaction mixture contained 0.75 μmol/L of *Taq* polymerase (2×Rapid *Taq* Master Mix, Vazyme,China).

For 410 bp fragment, the PCR cycle had the following parameters: 95°C for 5 min, 35 cycles at 94 °C for 30s, 60 °C for 30s, 72°C for 60s, and a final step at 72°C for 10 min.

For 392 bp fragment, The PCR cycle had the following parameters: 95°C for 5 min, 35 cycles at 94 °C for 30s, 55 °C for 30 s, 72 °C for 60s,and a final step at 72°C for 10 min.

Characteristics of amplified fragments and corresponding primer sequences

Targeted DNA	Gene	Primer sequence (5'-3')	Fragment	Cycling conditions of PCR assays	Reference
identification of rodent species	*cytb*	Forward: (5'-ACCAATGACATGAAAAATCATCGTT-3')	1242 bp	95°C for 5 min, 35 cycles at 94°C for 30s, 55°C for 60s, 72°C for 60s, and a final step at 72°C for 10 min.	[1]
		Reverse: (5'-TCTCCATTTCTGGTTTACAAGAC-3')			
		Forward: (5'-TAGAATAYCAGCTTTGGGTG-3')	1200 bp	94°C for 3 min, 35 cycles at 94°C for 35s, 53°C for 35s, 72°C for 80s, and a final step at 72°C for 10 min.	[2]
		Reverse: (5'-CGAAGCTTGATAYGAAAAAYCAYYGTTG-3')			
identification of bird species	*16sRNA*	Forward (5'-CTGTAGGCCTTTAAGCAGC-3')	910 bp	95°C for 5 min, 35 cycles at 95°C for 30s, 55°C for 40 s, 72°C for 60s, and a final step at 72°C for 10 min.	[3]
		Reverse (5'-AGGATGTCCTGATCCAACAT-3')			
	D-loop-mtDNA	Forward (5'-TCACGTGAAATCAGCAACCC-3')	924 bp	94°C for 3 min, 35 cycles at 94°C for 30s, 59°C for 30s, 72°C for 30s, and a final step at 72°C for 10 min.	[4]
		Reverse			

		(5'-CATCTTCAGTGCCATGCTTT-3')			
identification of lizard species	*COX1*	Forward (5' -TYTCWACWAAYCAYAAAGAYATCGG-3')	650 bp	94°C for 3 min, 40 cycles at 94°C for 30s, 50°C for 30s, 72°C for 30s, and a final step at 72°C for 10 min.	[5]
		Reverse (5'-ACYTCRGGRTGRCCRAARAATCA-3')			
E. bieneusi	*ITS*	Forward 1: (5'-GGTCATAGGGATGAAGAG-3')	410 bp	95°C for 5 min, 35 cycles at 94 °C for 30s, 60°C for 30s, 72°C for 60s, and a final step at 72°C for 10 min.	[6]
		Reverse 1: (5'-GGTCATAGGGATGAAGAG-3')			
		Forward 2: (5'-GCTCTGAATATCTATGGCT-3')	392 bp	95°C for 5 min, 35 cycles at 94°C for 30s, 55°C for 30s, 72°C for 60s,and a final step at 72°C for 10 min.	
		Reverse 2: (5'-ATCGCCGACGGATCCAAGTG-3')			

[1] Hu Qun, Ma Sijie, Zou Chunying, Tong Shumei. Species indentification of suspected samples of rats found in imported containers based on Cytb gene. Chinese Frontier Health Quarantine Feb.2014,Vol 37,No.1.

[2] Chen, Jin-Tao., Qin, Jing., Li, Kun., Xu, Qi-Yi., Wang, Xiao-Ping. Identification and characterization of a novel subtype of Tula virus in *Microtus arvalis* obscurus voles sampled from Xinjiang, China. Infection, genetics and evolution: journal of molecular epidemiology and evolutionary genetics in infectious diseases, 2019, 75:104012.

[3] Liu Dawei, Ying Gengdi, Zhou Yongwu, Fei Yiling, et al. Identification of a Bird Remain in a Case of Destroying Wildlife Resources Based on the COI and 16sRNA Genes［J］. Chinese Journal of Wildlife, 2022,43(3):715-724.

[4] Crochet PA, Desmarais E. Slow rate of evolution in the mitochondrial control region of gulls (Aves: Laridae). Mol Biol Evol. 2000 Dec;17(12):1797-806. doi: 10.1093/oxfordjournals.molbev.a026280..

[5] Che J, Chen HM, Yang JX, Jin JQ, Jiang K, Yuan ZY, Murphy RW, Zhang YP. Universal COI primers for DNA barcoding amphibians. Mol Ecol Resour. 2012 Mar;12(2):247-58. doi: 10.1111/j.1755-0998.2011.03090.x.

[6] Buckholt, Michael A., Lee, John H., Tzipori, Saul.. Prevalence of *Enterocytozoon bieneusi* in swine: an 18-month survey at a slaughterhouse in Massachusetts. Applied and environmental microbiology, 2002, 68(5):2595-9.
