# Supplementary figures and images for: Genetic diversity of Enterocytozoon bieneusi in 1099 wild animals and 273 imported pastured donkeys in northern China
Source: Parasit Vectors. 2025 Mar 13;18:105. doi: 10.1186/s13071-025-06739-6 (PMC11905730; doi:10.1186/s13071-025-06739-6)

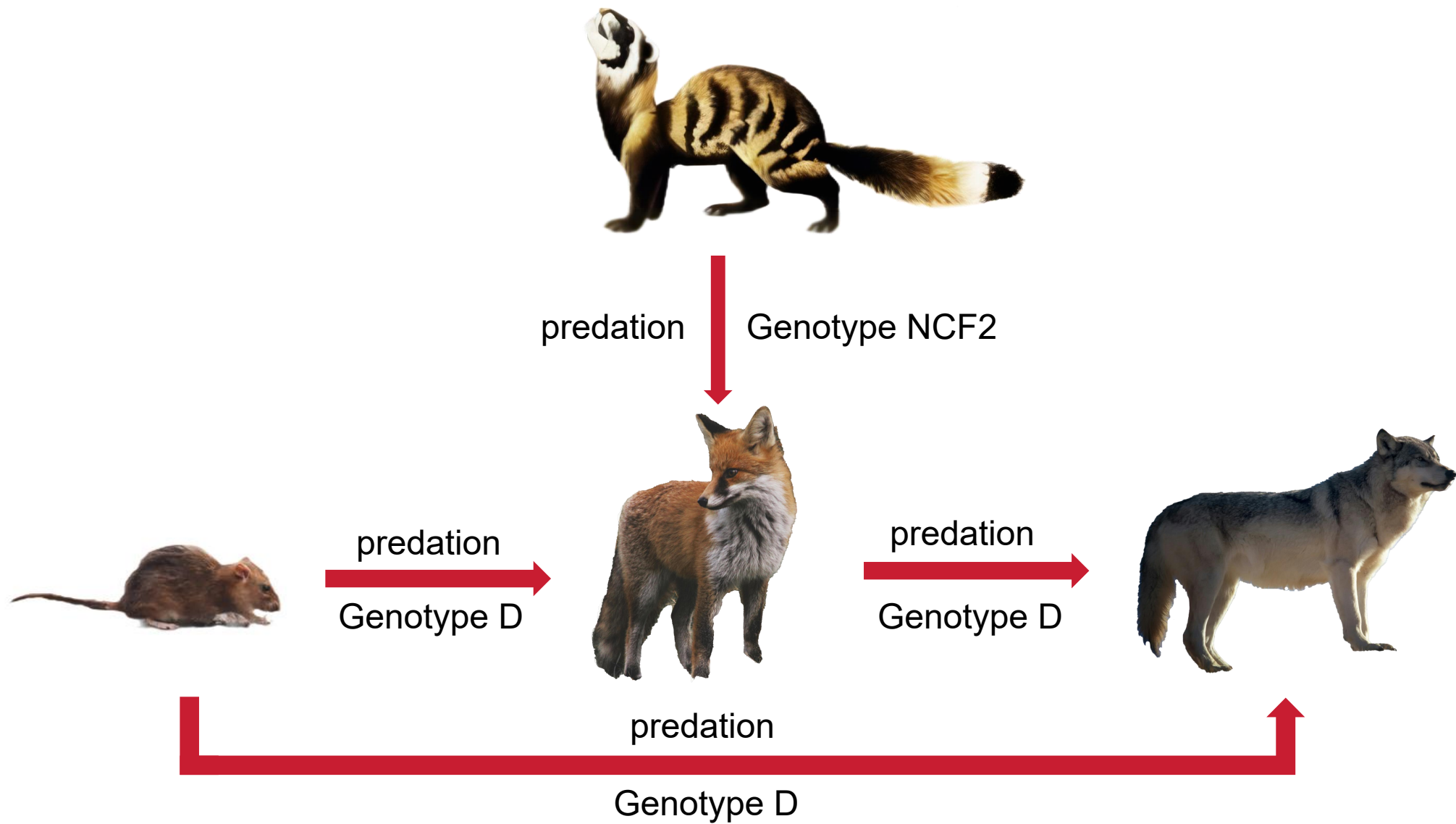

Supplement: Supplementary file 4 — Additional file 4: In this study, transmission routes involving the predator-prey relationship between hosts of genotype D and genotype NCF2 were investigated. [file 13071_2025_6739_MOESM4_ESM.pdf]

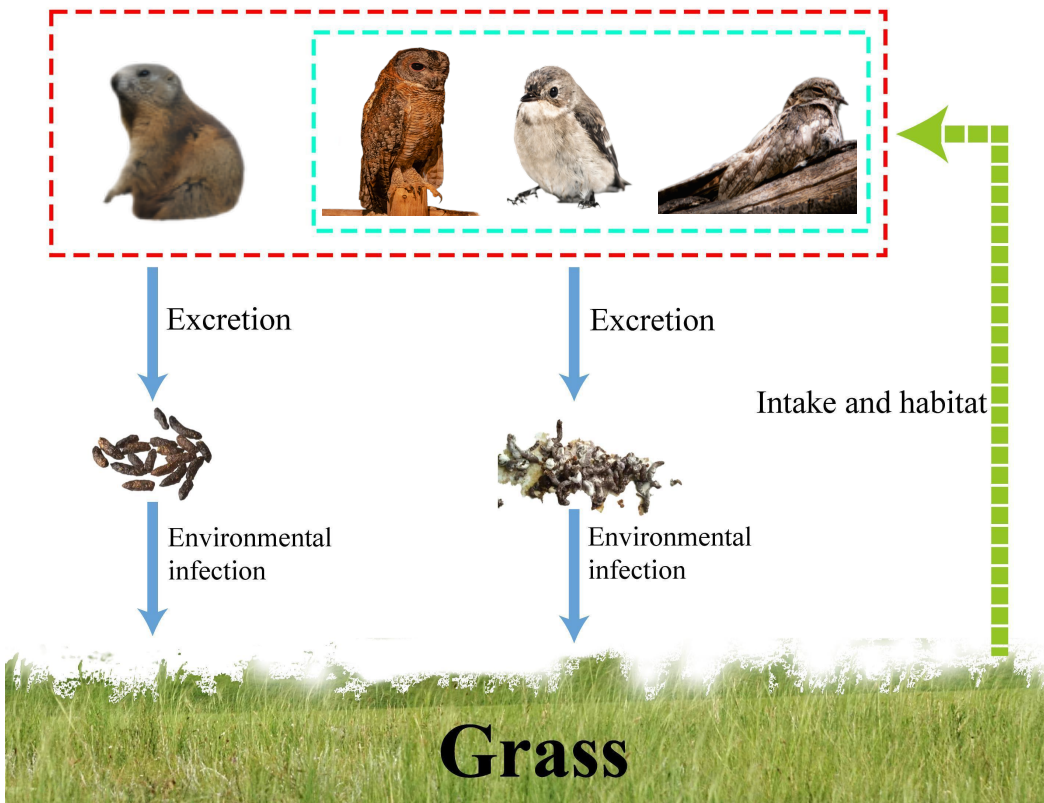

Supplement: Supplementary file 5 — Additional file 5: Genotype horse1 was detected in Himalayan marmots, owls, jungle nightjars and Hume’s groundpeckers, revealing habitat overlap in these animals. [file 13071_2025_6739_MOESM5_ESM.pdf]
